# Supplementary material for: Suppression of the Peripheral Immune System Limits the Central Immune Response Following Cuprizone-Feeding: Relevance to Modelling Multiple Sclerosis
Source: Cells. 2019 Oct 24;8(11):1314. doi: 10.3390/cells8111314 (PMC6912385; doi:10.3390/cells8111314)
Supplement: Supplementary file 1 [file cells-08-01314-s001.pdf]

Suppression of the peripheral immune system limits the central immune response following cuprizone-feeding: Relevance to modelling Multiple Sclerosis

Monokesh K. Sen[1], Mohammed S.M. Almuslehi[1,2], Erika Gyengesi[1], Simon J. Myers[3], Peter J. Shortland[3], David A. Mahns[1*], and Jens R. Coorssen[4*]

[1]School of Medicine, Western Sydney University, Locked Bag 1797, Penrith, NSW 2751, Australia; monokesh.sen@westernsydney.edu.au.
[1,2]School of Medicine, Western Sydney University, Locked Bag 1797, Penrith, NSW 2751, Australia and [2]Department of Physiology, College of Veterinary Medicine, Diyala University, Diyala, Iraq; m.almuslehi@westernsydney.edu.au.
[1]School of Medicine, Western Sydney University, Locked Bag 1797, Penrith, NSW 2751, Australia; e.gyengesi@westernsydney.edu.au.
[1]School of Medicine, Western Sydney University, Locked Bag 1797, Penrith, NSW 2751, Australia; d.mahns@westernsydney.edu.au.
[3]School of Science and Health, Western Sydney University, Locked Bag 1797, Penrith, NSW 2751, Australia; s.myers@westernsydney.edu.au.
[3]School of Science and Health, Western Sydney University, Locked Bag 1797, Penrith, NSW 2751, Australia; p.shortland@westernsydney.edu.au.
[4]Department of Health Sciences, Faculty of Applied Health Sciences, and Department of Biological Sciences, Faculty of Mathematics and Science, Brock University, St. Catharines, Ontario, ON L2S 3A1, Canada; jcoorssen@brocku.ca

***Co-corresponding authors**
[1]School of Medicine, Western Sydney University, Locked Bag 1797, Penrith, NSW 2751, Australia; d.mahns@westernsydney.edu.au.

[4]Department of Health Sciences, Faculty of Applied Health Sciences, and Department of Biological Sciences, Faculty of Mathematics and Science, Brock University, St. Catharines, Ontario, ON L2S 3A1, Canada; jcoorssen@brocku.ca.

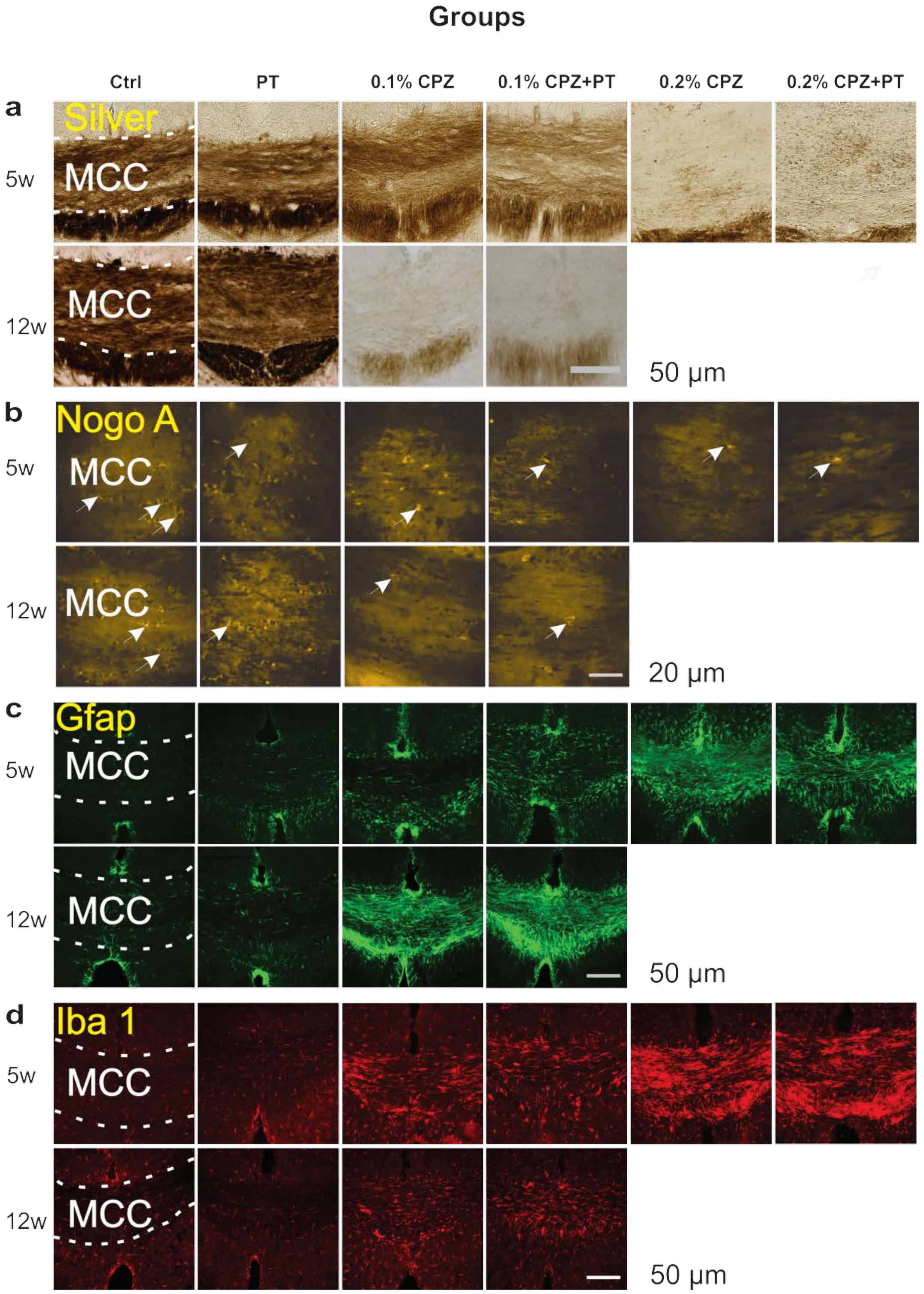

Figure 1: Histological effects of different treatments on the midline corpus callosum
Representative images of silver-stained brain sections from 5- and 12- week experimental groups to assess demyelination (**a**), or immunohistochemistry to identify the cell bodies of mature oligodendrocytes using Nogo A (**b**), astrocytes using Gfap (**c**) or microglia using Iba 1 (**d**) antibodies. Dashed lines represent the upper and lower borders of the MCC. Arrows indicate Nogo A positive cells.

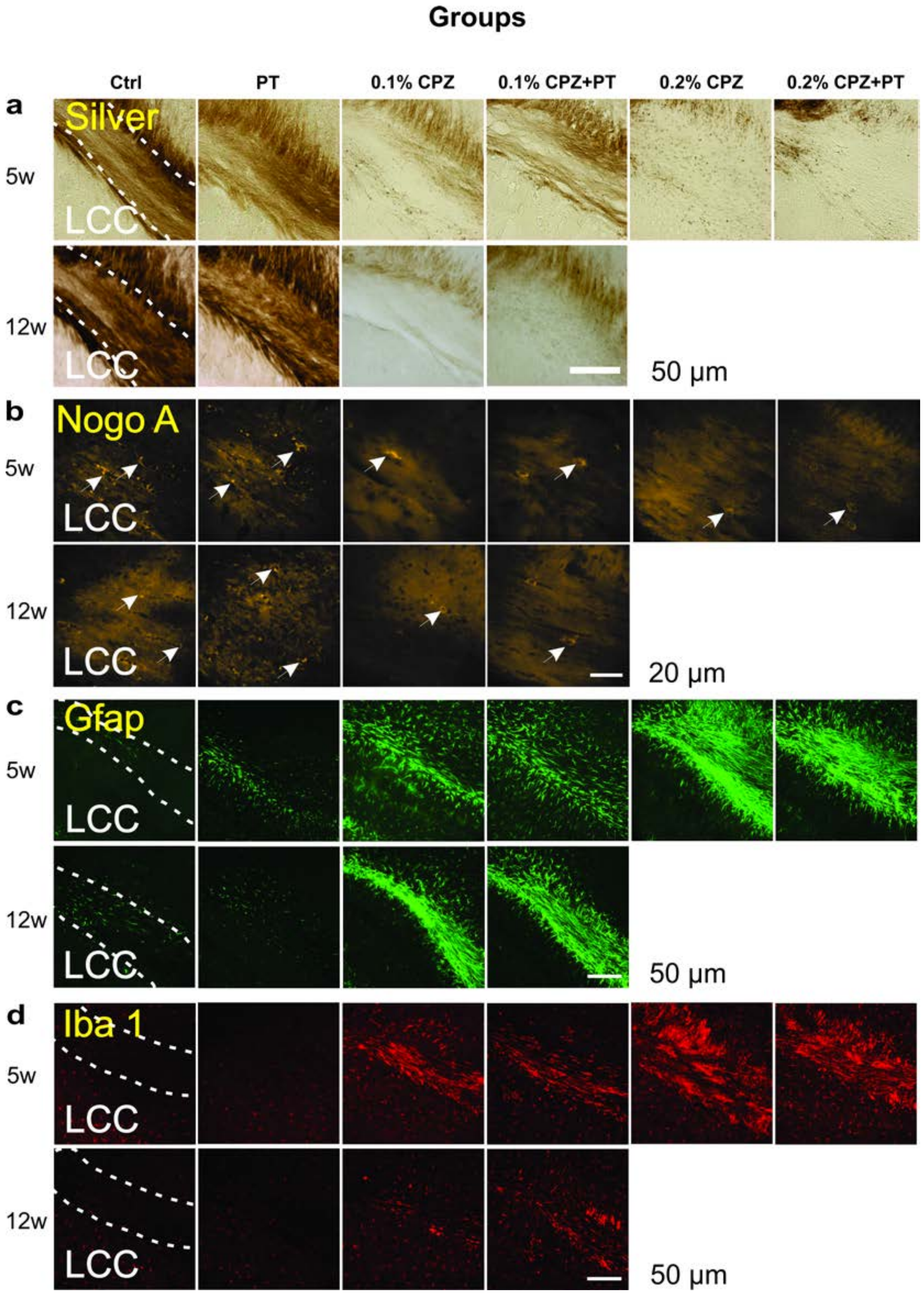

Figure 2: Histological effects of different treatments on the lateral corpus callosum
Representative images of silver-stained brain sections from 5- and 12- week experimental groups to assess demyelination (**a**), or immunohistochemistry to identify the cell bodies of mature oligodendrocytes using Nogo A (**b**), astrocytes using Gfap (**c**) or microglia using Iba 1 (**d**) antibodies. Dashed lines represent the upper and lower borders of the LCC. Arrows indicate Nogo A positive cells.

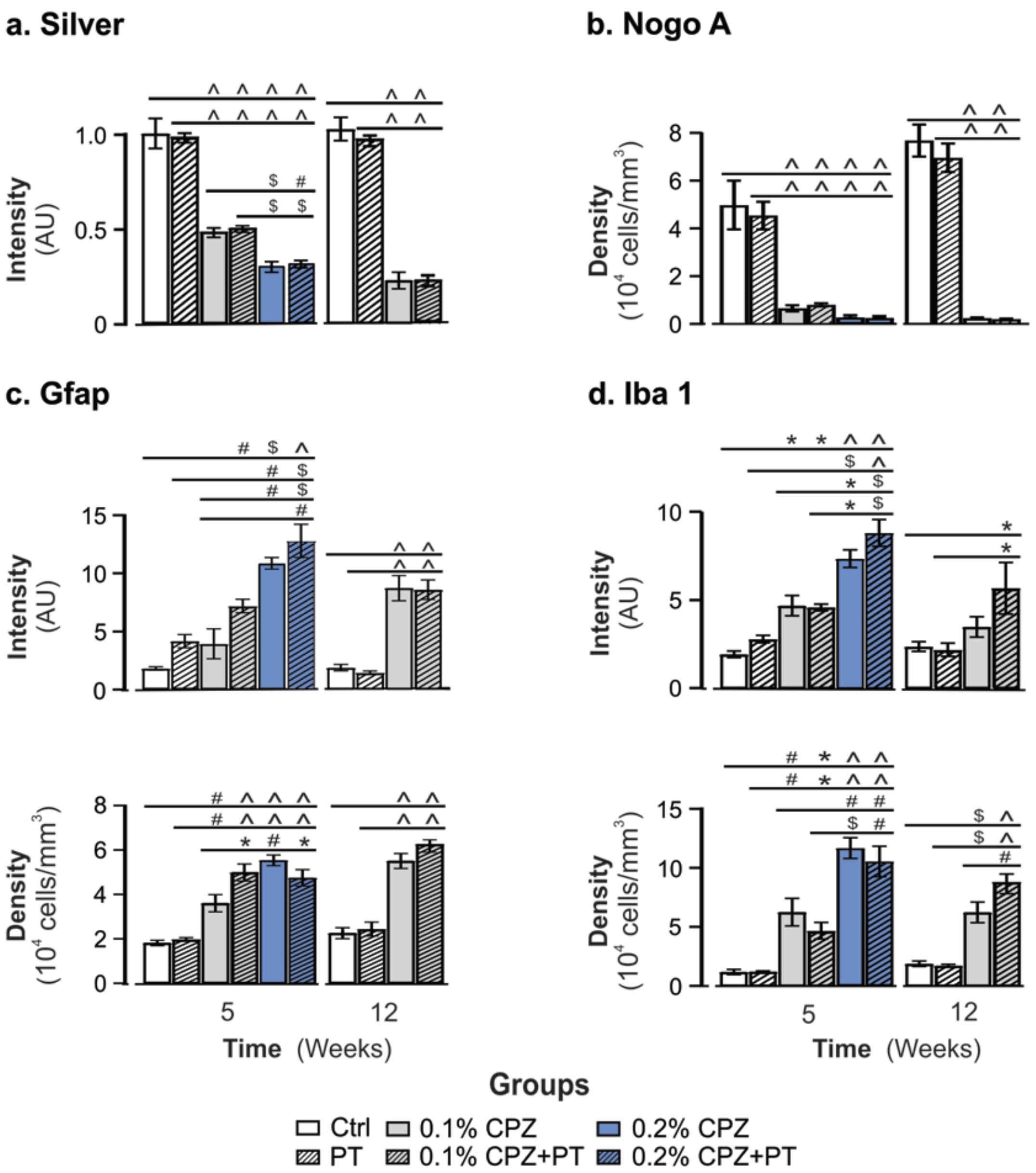

Figure 3: Quantification of demyelination, cell death and gliosis in lateral corpus callosum
a) Silver staining. CPZ-feeding(±PT) led to significant demyelination and reduced silver staining intensity at 5 and 12 weeks in the LCC. PT alone had no effect. Feeding 0.1% CPZ for longer (12 weeks) produced a comparable demyelination to that seen with 0.2% for 5 weeks. **b**) Nogo A. CPZ-feeding(±PT) led to significant oligodendrocytosis with 0.1% CPZ as effective as 0.2% CPZ at either time point. PT alone had no effect. **c**) Gfap. Staining intensity increased in a dose dependent fashion the CPZ(±PT) treated groups and this was associated with an increase the number of Gfap positive astrocytes at both time points. PT only did not evoke a Gfap response. **d**) Iba 1. Increased Iba 1 fluorescence intensity and number of Iba 1 positive microglia were seen in both 5-and 12-week groups. Significant effects were best seen at the highest doses used at each time point. PT only produced no microglial response. Data are presented as mean ± SEM. One-way ANOVA and Tukey post hoc analysis was used to determine differences among groups (*p<0.05, #p<0.01, \$$p$<0.001 and ^$p$<0.0001). Quantitation based on analysis of 5-9 sections/animal, 3-5 animals/group.

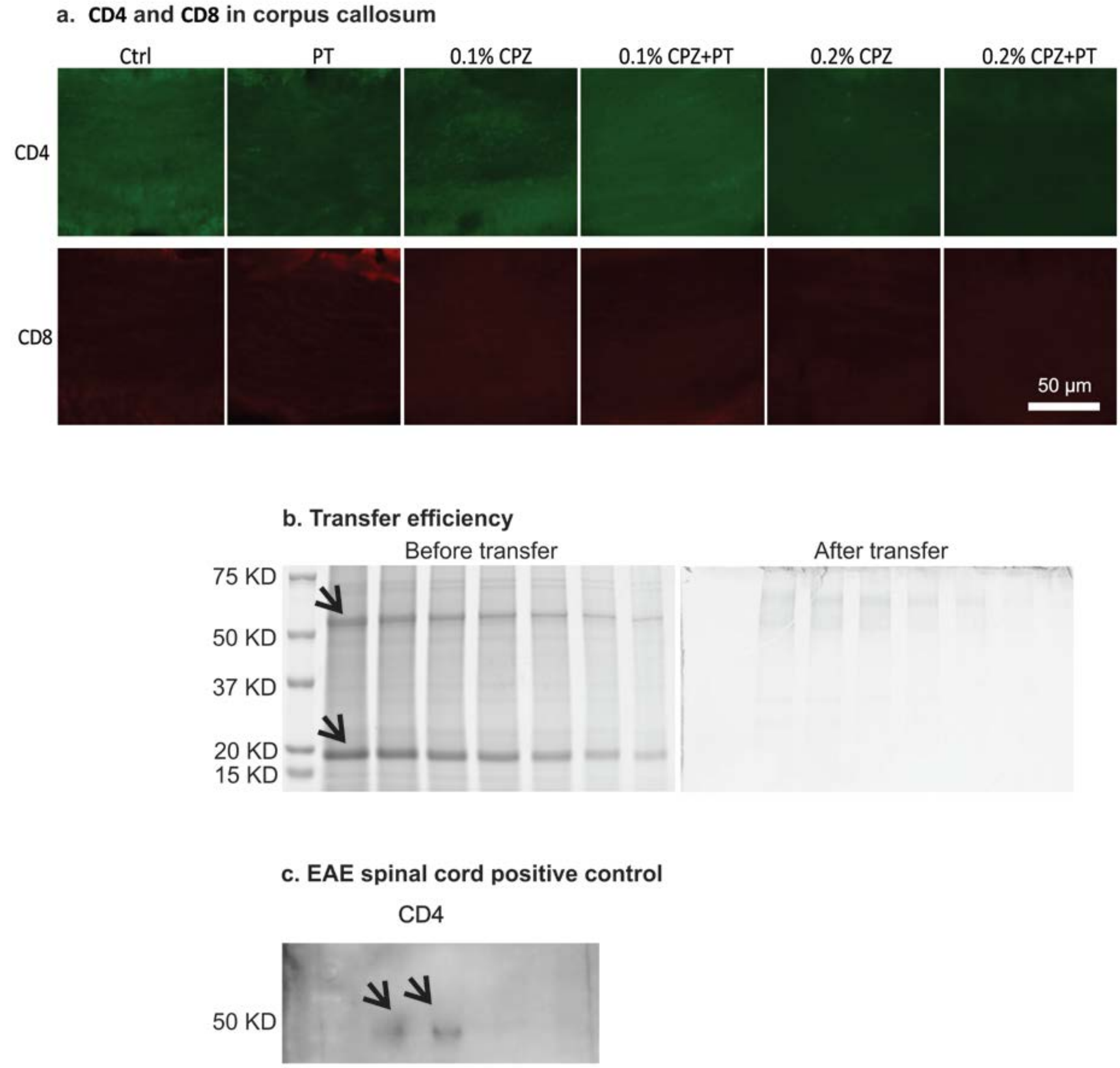

Figure 4: Immune staining and transfer efficiency

a) Immunofluorescence staining of CD4 and CD8 in the corpus callosum from the 5-week study (n=5 sections/animal, n=3 animals/group). No positive cells were detected. **b**) 1D gels used to quantify the transfer efficiency of respective regions of CD4 (50 KD) and CD8 (30 KD) proteins from gel to PVDF membrane (n=3 bands/gel, n=2 gels). Arrows indicate the bands quantified. **c**) 40 μg spinal cord homogenate was used to detect CD4 signal from EAE (n=2 animals, n=3 gels). Arrows indicate the CD4 band.

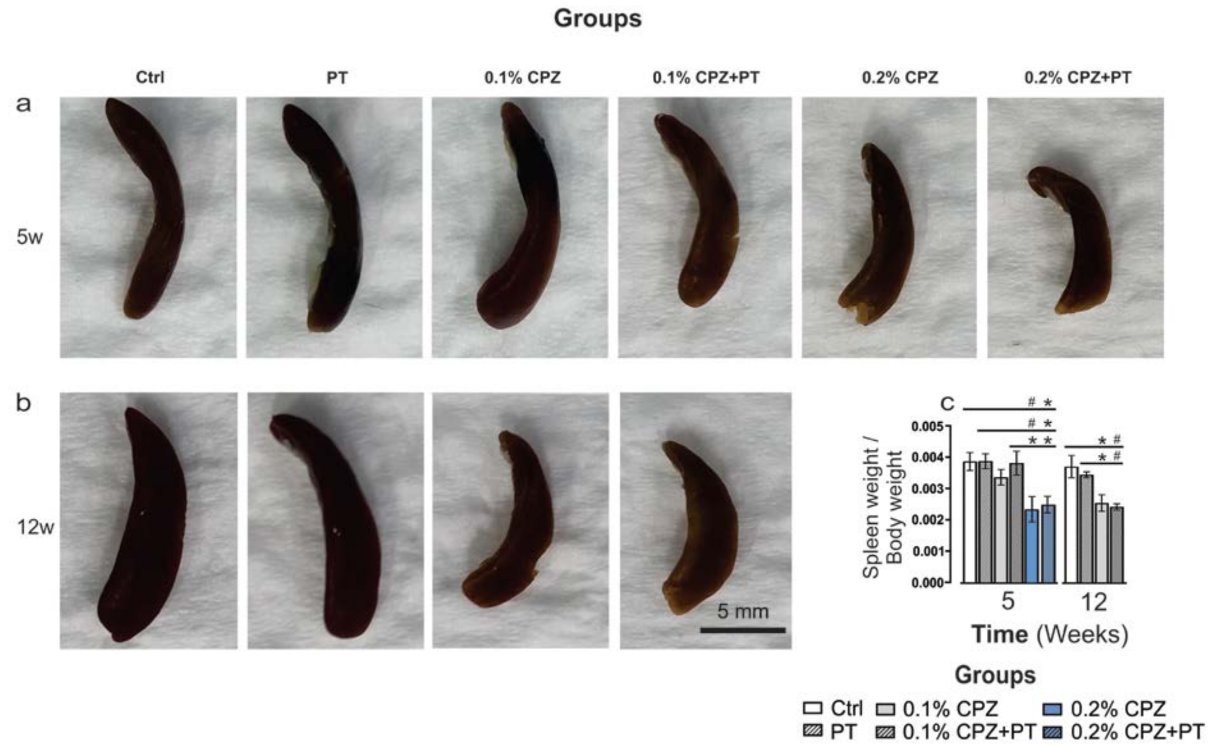

Figure 5: Dose and time dependent splenic atrophy

Representative images (**a** and **b**) of spleen from 5- and 12-week studies, respectively. Scale bar is 5 mm. Normalized (spleen tissue mass/body weight) splenic mass is shown in **c**. Feeding with 0.2% CPZ(±PT) for 5 weeks or 0.1% CPZ(±PT) for 12 weeks resulted in a significant reduction of splenic mass. Data are presented as mean ± SEM. One-way ANOVA and Tukey post hoc analysis was used to determine differences among groups (*p<0.05 and #p<0.01). Quantitation based on analysis of 3 and 5 spleens from 5 and 12 weeks, respectively.

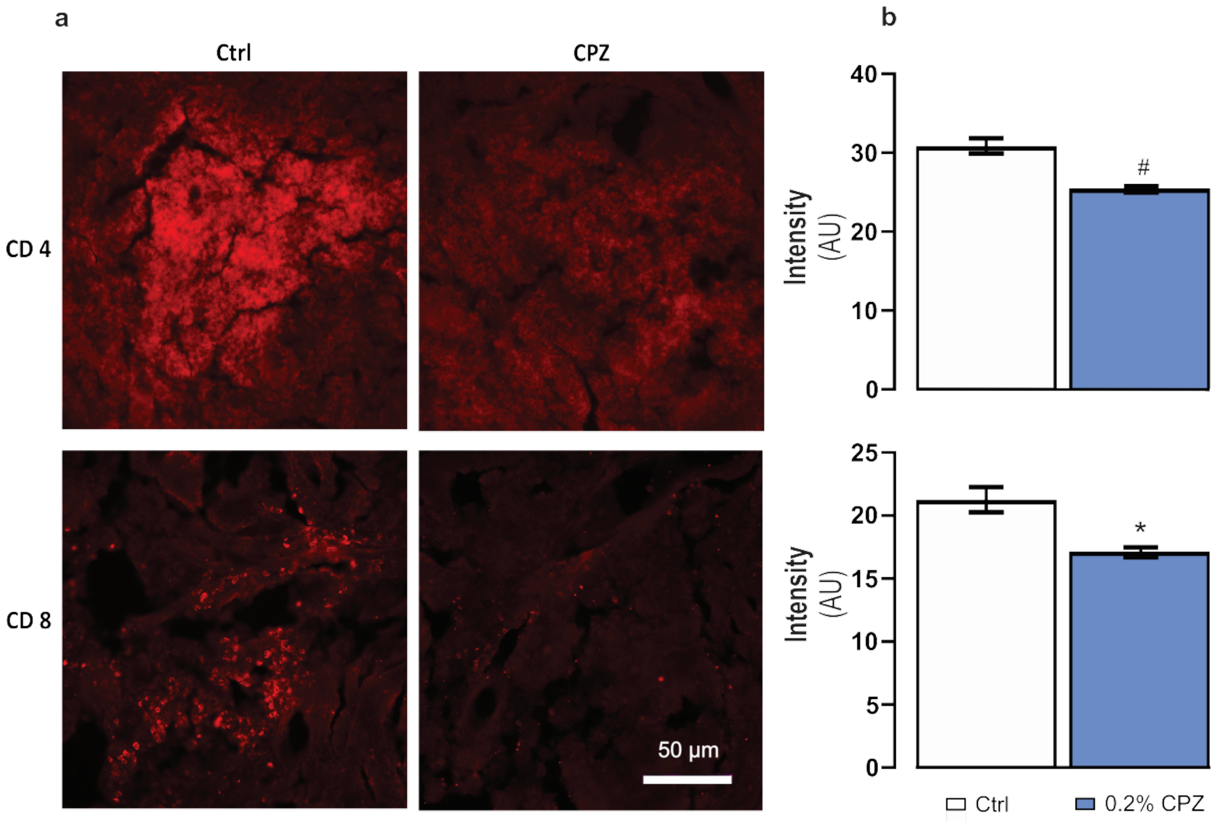

Figure 6: Reduction of T-cell fluorescence intensity

Representative images of spleen (**a)** and quantification (**b**) of CD4 and CD8 from Ctrl and 0.2% CPZ, respectively. Scale bar is 50 µm. Feeding with 0.2% CPZ showed a significant reduction of splenic CD4 and CD8 T-cells. Data are presented as mean ± SEM. Unpaired two-tailed t test was used to determine differences between groups (*$p<0.05$ and #$p<0.01$). Quantitation based on analysis of 3 spleens/group and 10 sections/spleen.

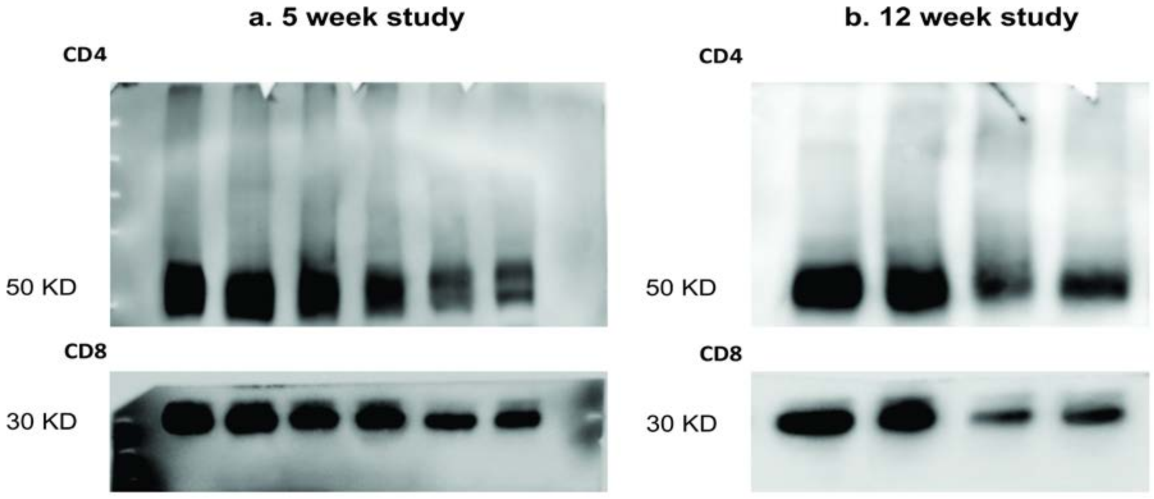

Figure 7: Western blot images of CD4 and CD8 from spleen samples from both 5- and 12-week studies

Table 1: Quantification of the total number of protein spots in 2D gels of whole brain soluble and membrane proteomes

Tissue fraction	Ctrl	PT	0.1% CPZ	0.1% CPZ +PT	0.2% CPZ	0.2% CPZ + PT	Feeding duration
**SP**	829±6	830±15	866±12	871±19	860±12	841±12	5 weeks
**MP**	766±10	802±12	798±15	835±21	779±13	778±16	
**SP**	808±6	802±2	791±5	789±4	-	-	12 weeks
**MP**	719±2	708 ±5	716 ±4	727±5	-	-	

Key: Soluble protein (SP) and membrane protein (MP), (n=5 animals/group, n=15 gels/fraction).

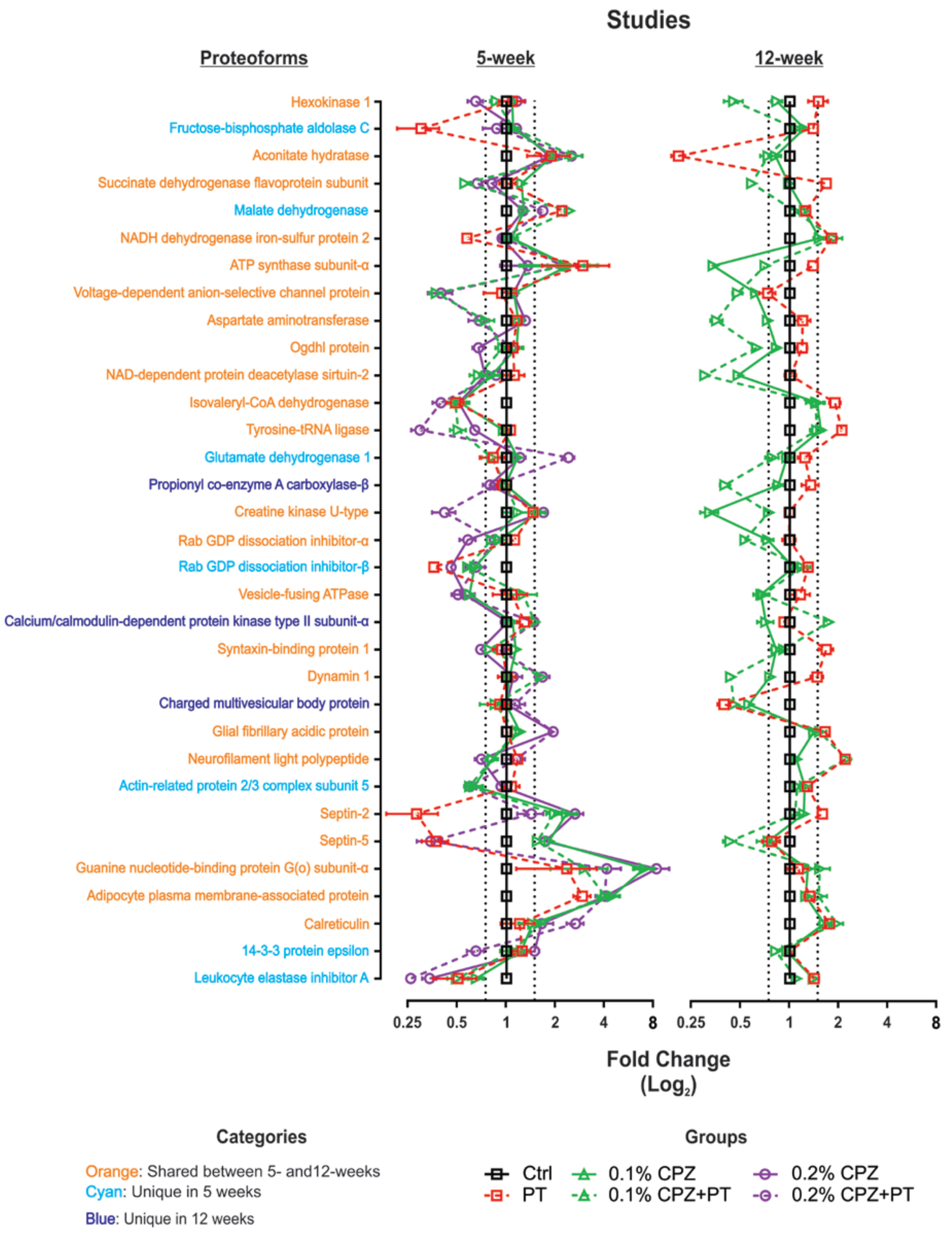

Figure 8: Pattern of proteoform change

Log2 fold changes in abundance for 33 proteoforms relative to Ctrl showing the pattern of changes (colour marked in left Y-axis) at both time points. Changes (increase or decrease) in abundance (≥1.5 fold) shared at both 5 and 12 weeks are shown in orange. Abundance changes observed following 5 weeks (≥1.5 fold) but not 12 weeks (<1.5 fold) of CPZ-feeding are shown in cyan. Proteoforms unchanged at 5 weeks (<1.5 fold) but changed after 12 weeks (≥1.5 fold) are shown in blue.

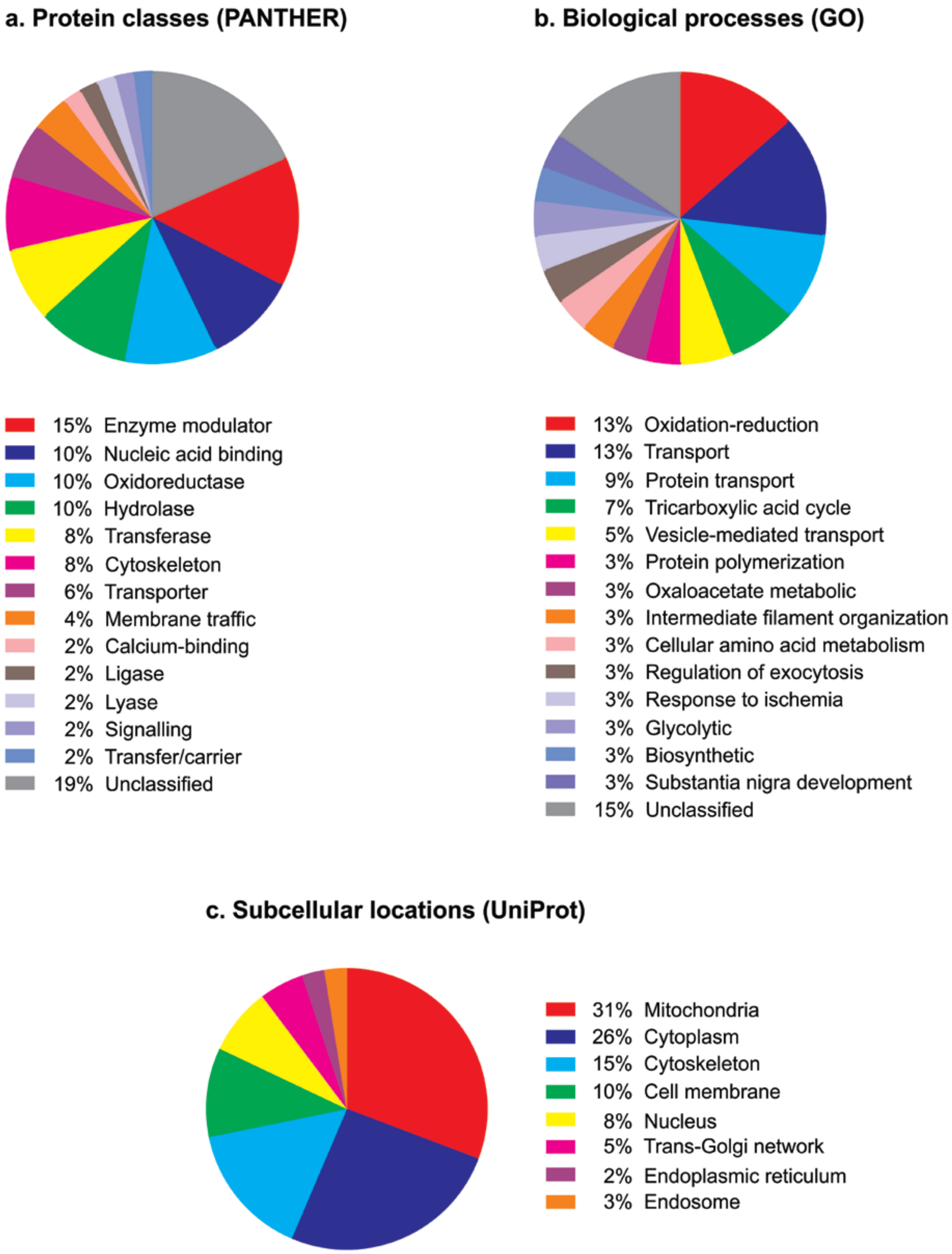

Figure 9: Functional clustering
Pie charts show the distribution of proteins according to **a**) Protein classes (characterized using PANTHER), **b**) Biological processes (categorised using GO) and **c**) Subcellular locations (categorised using UniProt).

a

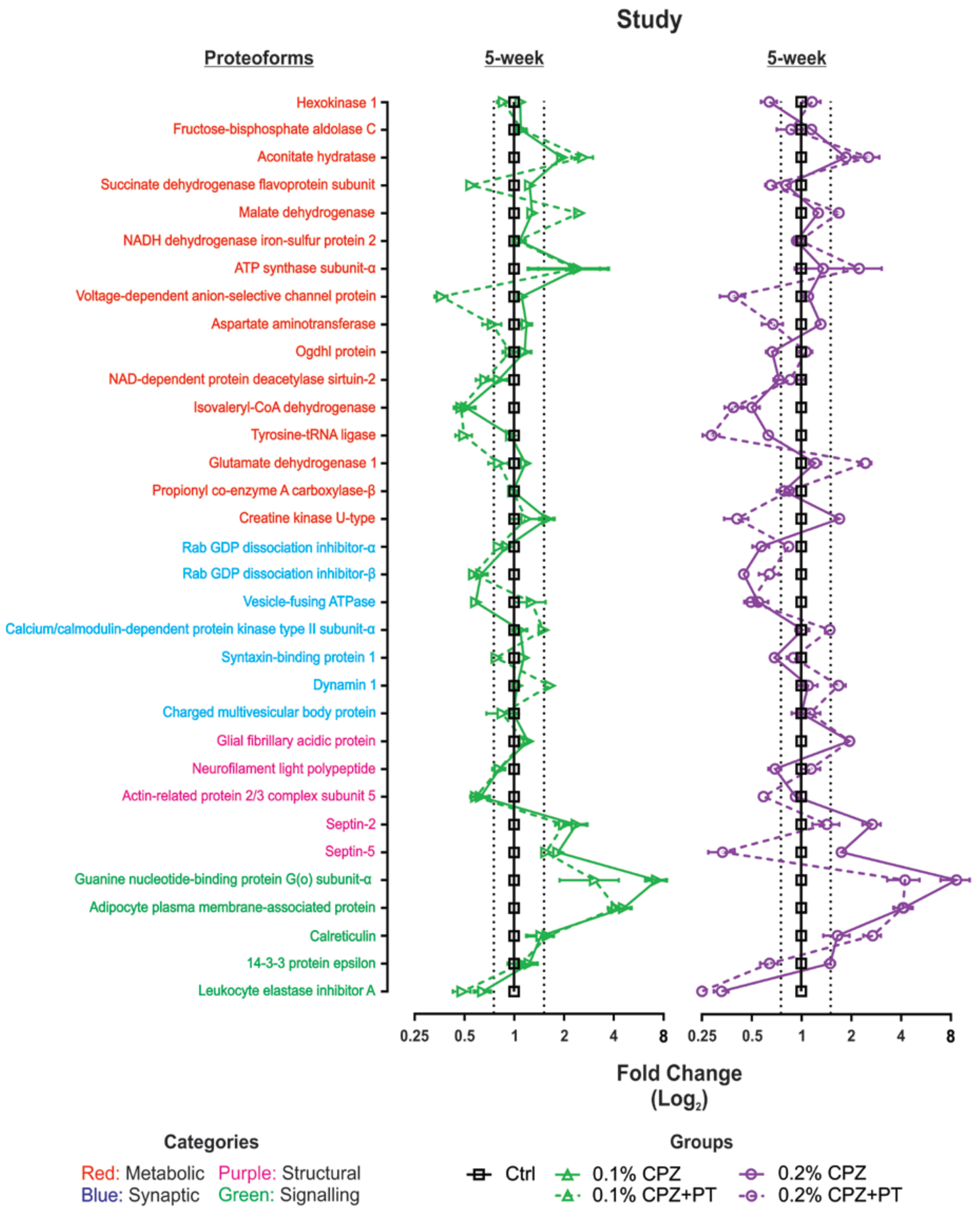

Study
Proteoforms
5-week
5-week
Hexokinase 1
Fructose-bisphosphate aldolase C
Aconitate hydratase
Succinate dehydrogenase flavoprotein subunit
Malate dehydrogenase
NADH dehydrogenase iron-sulfur protein 2
ATP synthase subunit-α
Voltage-dependent anion-selective channel protein
Aspartate aminotransferase
Ogdhl protein
NAD-dependent protein deacetylase sirtuin-2
Isovaleryl-CoA dehydrogenase
Tyrosine-tRNA ligase
Glutamate dehydrogenase 1
Propionyl co-enzyme A carboxylase-β
Creatine kinase U-type
Rab GDP dissociation inhibitor-α
Rab GDP dissociation inhibitor-β
Vesicle-fusing ATPase
Calcium/calmodulin-dependent protein kinase type II subunit-α
Syntaxin-binding protein 1
Dynamin 1
Charged multivesicular body protein
Glial fibrillary acidic protein
Neurofilament light polypeptide
Actin-related protein 2/3 complex subunit 5
Septin-2
Septin-5
Guanine nucleotide-binding protein G(o) subunit-α
Adipocyte plasma membrane-associated protein
Calreticulin
14-3-3 protein epsilon
Leukocyte elastase inhibitor A
0.25 0.5 1 2 4 8
0.25 0.5 1 2 4 8
Fold Change
(Log2)
Categories
Red: Metabolic Purple: Structural
Blue: Synaptic Green: Signalling
Groups
Ctrl
0.1% CPZ
0.1% CPZ+PT
0.2% CPZ
0.2% CPZ+PT

b

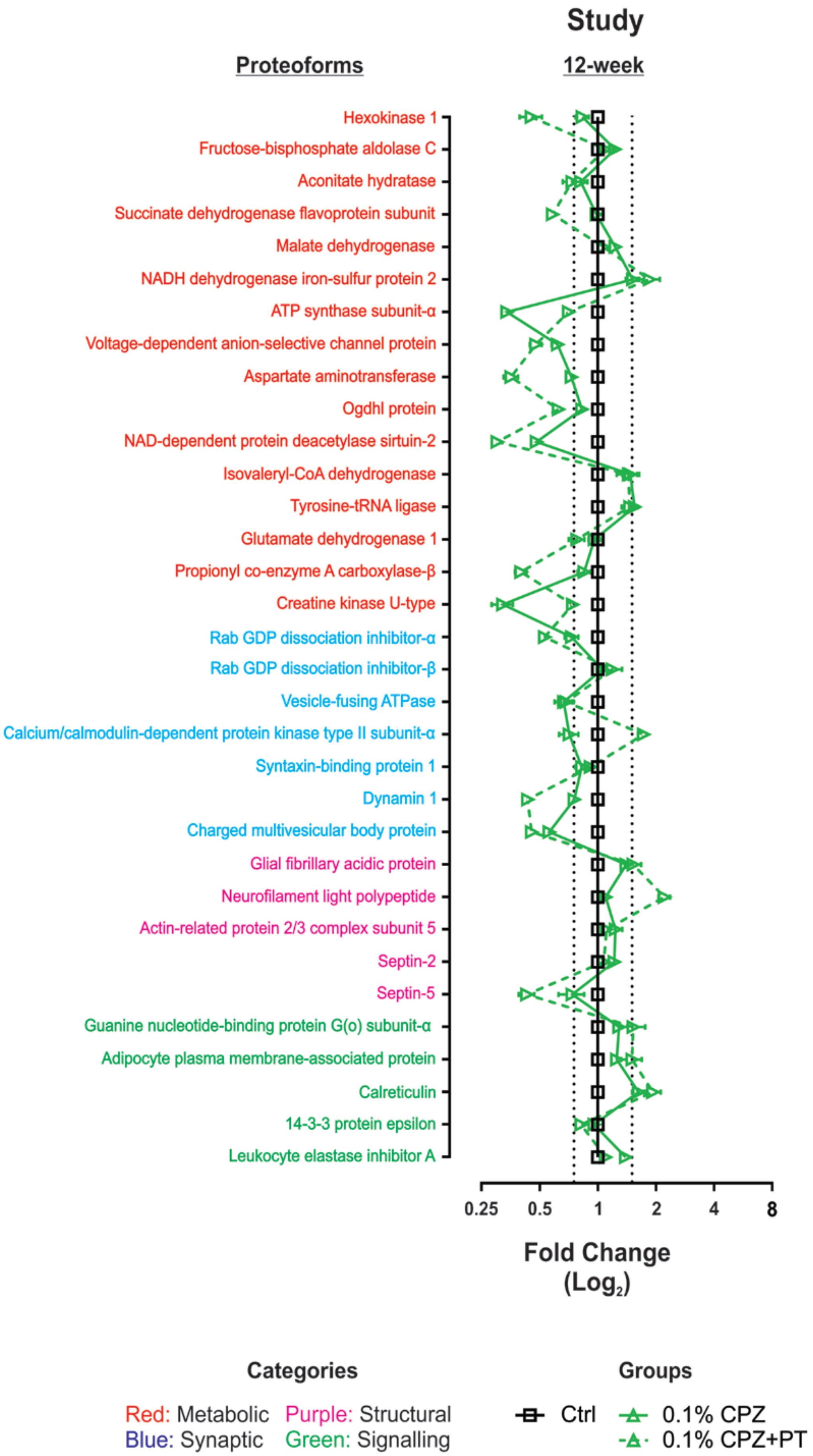

Study
Proteoforms
12-week
Hexokinase 1
Fructose-bisphosphate aldolase C
Aconitate hydratase
Succinate dehydrogenase flavoprotein subunit
Malate dehydrogenase
NADH dehydrogenase iron-sulfur protein 2
ATP synthase subunit-α
Voltage-dependent anion-selective channel protein
Aspartate aminotransferase
Ogdhl protein
NAD-dependent protein deacetylase sirtuin-2
Isovaleryl-CoA dehydrogenase
Tyrosine-tRNA ligase
Glutamate dehydrogenase 1
Propionyl co-enzyme A carboxylase-β
Creatine kinase U-type
Rab GDP dissociation inhibitor-α
Rab GDP dissociation inhibitor-β
Vesicle-fusing ATPase
Calcium/calmodulin-dependent protein kinase type II subunit-α
Syntaxin-binding protein 1
Dynamin 1
Charged multivesicular body protein
Glial fibrillary acidic protein
Neurofilament light polypeptide
Actin-related protein 2/3 complex subunit 5
Septin-2
Septin-5
Guanine nucleotide-binding protein G(o) subunit-α
Adipocyte plasma membrane-associated protein
Calreticulin
14-3-3 protein epsilon
Leukocyte elastase inhibitor A
0.25
0.5
1
2
4
8
Fold Change
(Log2)
Categories
Red: Metabolic
Purple: Structural
Blue: Synaptic
Green: Signalling
Groups
Ctrl
0.1% CPZ
0.1% CPZ+PT

c

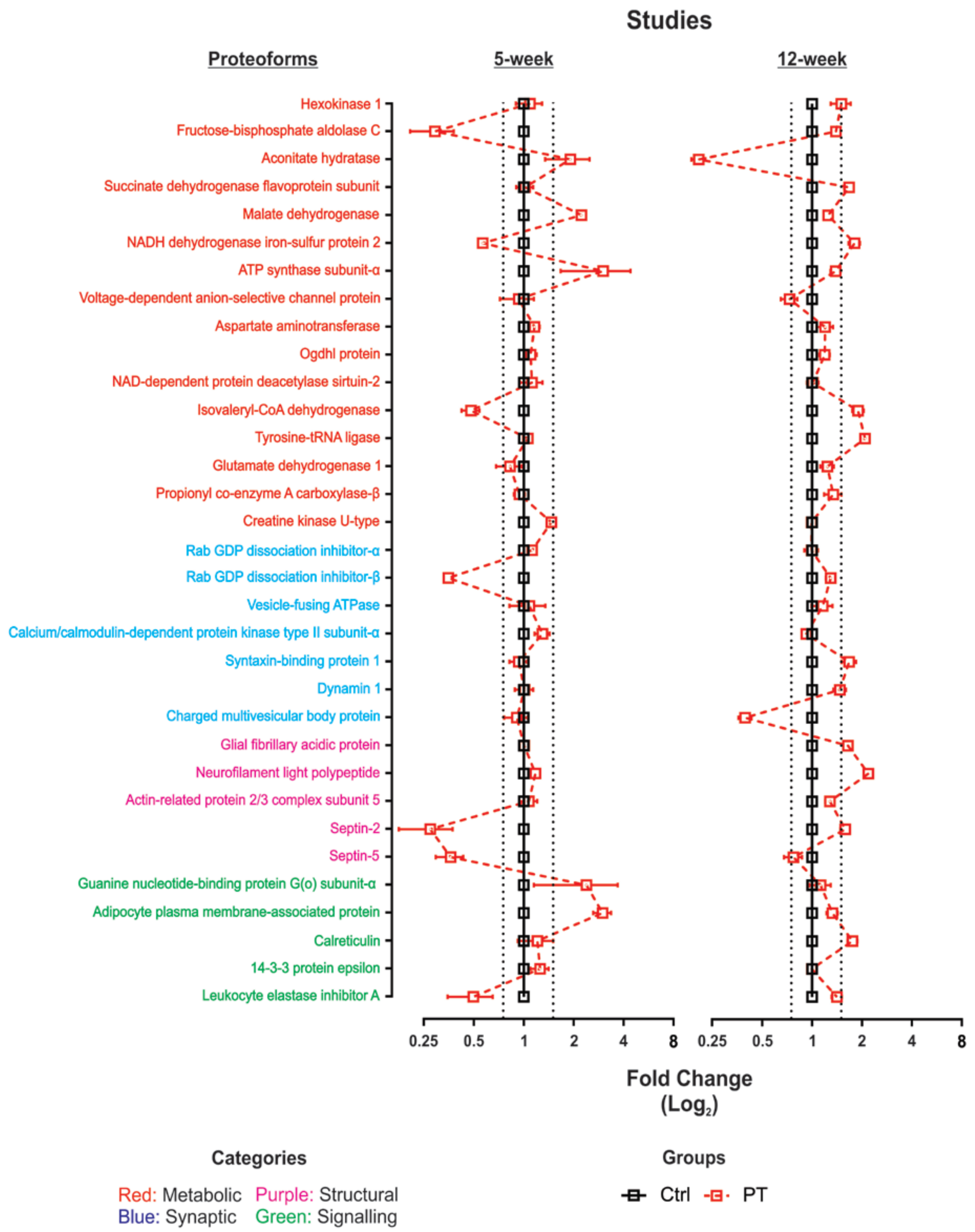

Figure 10: Duration and treatment-dependent proteoform changes

Log2 fold changes in abundance for 33 proteoforms relative to Ctrl showing the changes with CPZ (±PT) in the **a**) 5 week study or **b**) 12 week study. Changes only in PT groups at both 5 and 12 weeks are shown in **c**).

Table 2: Comparison of PT group to the PT+CPZ groups only

Protein name	5 weeks	12 weeks
Hexokinase 1	0.1PT=PT=0.2PT	0.1PT<PT
Fructose-bisphosphate aldolase C	0.1PT>PT<0.2PT	0.1PT=PT
Aconitate hydratase	0.1PT>PT<0.2PT	0.1PT>PT
Succinate dehydrogenase flavoprotein subunit	0.1PT<PT>0.2PT	0.1PT<PT
Malate dehydrogenase	0.1PT=PT>0.2PT	0.1PT=PT
NADH dehydrogenase iron-sulfur protein 2	0.1PT>PT<0.2PT	0.1PT=PT
ATP synthase subunit-α	0.1PT=PT=0.2PT	0.1PT<PT
Voltage-dependent anion-selective channel protein	0.1PT<PT>0.2PT	0.1PT=PT
Aspartate aminotransferase	0.1PT<PT>0.2PT	0.1PT<PT
Ogdhl protein	0.1PT=PT=0.2PT	0.1PT<PT
NAD-dependent protein deacetylase sirtuin-2	0.1PT>PT=0.2PT	0.1PT<PT
Isovaleryl-CoA dehydrogenase	0.1PT=PT=0.2PT	0.1PT<PT
Tyrosine-tRNA ligase	0.1PT<PT>0.2PT	0.1PT<PT
Glutamate dehydrogenase 1	0.1PT=PT<0.2PT	0.1PT<PT
Propionyl co-enzyme A carboxylase-β	0.1PT=PT=0.2PT	0.1PT<PT
Creatine kinase U-type	0.1PT=PT>0.2PT	0.1PT=PT
Rab GDP dissociation inhibitor-α	0.1PT<PT>0.2PT	0.1PT<PT
Rab GDP dissociation inhibitor-β	0.1PT=PT=0.2PT	0.1PT=PT
Vesicle-fusing ATPase	0.1PT=PT>0.2PT	0.1PT<PT
Calcium/calmodulin-dependent protein kinase type II subunit-α	0.1PT=PT<0.2PT	0.1PT=PT
Syntaxin-binding protein 1	0.1PT=PT=0.2PT	0.1PT<PT
Dynamin 1	0.1PT>PT<0.2PT	0.1PT<PT
Charged multivesicular body protein	0.1PT=PT=0.2PT	0.1PT=PT
Glial fibrillary acidic protein	0.1PT=PT<0.2PT	0.1PT=PT
Neurofilament light polypeptide	0.1PT<PT=0.2PT	0.1PT=PT
Actin-related protein 2/3 complex subunit 5	0.1PT<PT>0.2PT	0.1PT=PT
Septin-2	0.1PT>PT<0.2PT	0.1PT<PT
Septin-5	0.1PT>PT=0.2PT	0.1PT<PT
Guanine nucleotide-binding protein G(o) subunit-α	0.1PT=PT<0.2PT	0.1PT>PT
Adipocyte plasma membrane-associated protein	0.1PT>PT<0.2PT	0.1PT=PT
Calreticulin	0.1PT=PT<0.2PT	0.1PT=PT
14-3-3 protein epsilon	0.1PT=PT=0.2PT	0.1PT=PT
Leukocyte elastase inhibitor A	0.1PT=PT=0.2PT	0.1PT<PT

Key: Significant (*p*<0.05) increases and decreases relative to PT are indicated by < and > symbols, respectively. =, no significant difference; 0.1% CPZ+PT abbreviated to 0.1PT and 0.2% CPZ+PT to 0.2PT.
